# Spatial Gene Expression Analysis Reveals Characteristic Gene Expression Patterns of De Novo Neuroendocrine Prostate Cancer Coexisting with Androgen Receptor Pathway Prostate Cancer

**DOI:** 10.3390/ijms24108955

**Published:** 2023-05-18

**Authors:** Ryuta Watanabe, Noriyoshi Miura, Mie Kurata, Riko Kitazawa, Tadahiko Kikugawa, Takashi Saika

**Affiliations:** 1 Department of Urology, Ehime University Hospital, Ehime 791-0204, Japan; 2 Human Biology Division, Fred Hutchinson Cancer Center, Seattle, WA 98109, USA; 3 Department of Analytical Pathology, Ehime University Graduate School of Medicine, Ehime 791-0295, Japan; 4 Division of Pathology, Proteo-Science Center, Ehime 790-0826, Japan; 5 Division of Diagnostic Pathology, Ehime University Hospital, Ehime 791-0204, Japan; riko@m.ehime-u.ac.jp

**Keywords:** AR pathway prostate cancer, CytAssist Visium, de novo NEPC, gene expression, simultaneous occurrence, spatial transcriptomics, treatment-induced neuroendocrine prostate cancer

## Abstract

Neuroendocrine prostate carcinoma (NEPC) accounts for less than 1% of prostate neoplasms and has extremely poorer prognosis than the typical androgen receptor pathway-positive adenocarcinoma of the prostate (ARPC). However, very few cases in which de novo NEPC and APRC are diagnosed simultaneously in the same tissue have been reported. We report herein a 78-year-old man of de novo metastatic NEPC coexisting with ARPC treated at Ehime University Hospital. Visium CytAssist Spatial Gene Expression analysis (10× genetics) was performed using formalin-fixed, paraffin-embedded (FFPE) samples. The neuroendocrine signatures were upregulated in NEPC sites, and androgen receptor signatures were upregulated in ARPC sites. TP53, RB1, or PTEN and upregulation of the homologous recombination repair genes at NEPC sites were not downregulated. Urothelial carcinoma markers were not elevated. Meanwhile, Rbfox3 and SFRTM2 levels were downregulated while the levels of the fibrosis markers HGF, HMOX1, ELN, and GREM1 were upregulated in the tumor microenvironment of NEPC. In conclusion, the findings of spatial gene expression analysis in a patient with coexisting ARPC and de novo NEPC are reported. The accumulation of cases and basic data will help with the development of novel treatments for NEPC and improve the prognosis of patients with castration-resistant prostate cancer.

## 1. Introduction

Neuroendocrine prostate carcinoma (NEPC) is a rare disease that accounts for less than 1% of prostate neoplasms [1] and has an extremely poorer prognosis than the typical androgen receptor pathway-positive adenocarcinoma of the prostate (ARPC). NEPC is the most lethal prostate cancer, characterized by resistance to hormone therapy, rapid progression, and visceral metastases. The average survival time of patients with NEPC is only 7 months [2]. Given that the disease progresses in an androgen-independent manner, ADT has little efficacy for this malignancy. There is also no standard chemotherapy specific for NEPC. Platinum-based chemotherapy agents such as cisplatin or carboplatin are typically used to treat NEPC based on the protocol for small cell lung cancer. However, their therapeutic effect is limited, and novel treatment options are urgently needed.

Although the disease can develop de novo, it occurs primarily after androgen deprivation therapy (ADT) [3]. Treatment-induced NEPC (t-NEPC) has recently received attention owing to the development of novel antiandrogen therapies However, although alterations in PTEN, TP53, RB1, and MYCN have been implicated in the development of NEPC. In a recent report by Chen et al. the most frequently mutated gene in NEPC was TP53 (49.8%); common copy number alterations in NEPC were RB1 loss (58.3%), TP53 loss (42.8%), PTEN loss (37.0%), AURKA amplification (28.2%), and MYCN amplification (22.9%) [4]. However, much remains to be learned about the genetic and molecular mechanisms underlying the development of t-NEPC. 

Given that prostate cancer is characterized by heterogeneity, its gene expression profile varies from site to site even within the same tissue. Therefore, conventional bulk gene analysis methods, which collectively analyze heterogeneous prostate tissues, have not been able to fully elucidate the genetic complexity of prostate cancer. It is also difficult to focus the analysis on cells of rare populations within the tumor microenvironment (TME) because of the loss of information on cell location. Further, the genetic mechanisms underlying the development of de novo NEPCs, as well as t-NEPCs, are unknown. The search for gene expression characteristic of de novo NEPCs by spatial transcriptomics may expand future options for targeted therapy.

This study aimed to examine the differences in spatial gene expression between de novo NEPC coexisting ARPC and their gene expression in the TME. To overcome the limitations of whole tumor analysis, we used the spatial gene expression analysis (CytAssist Visium) (10× genomics) technology. CytAssist Visium enabled to spatially detect and compare the gene expression of both de novo NEPC and ARPC in this coexisting case. 

## 2. Results

### 2.1. Localization of De Novo NEPC and ARPC and High-Expression Genes in the Prostate Tissue

**Figure 1:**
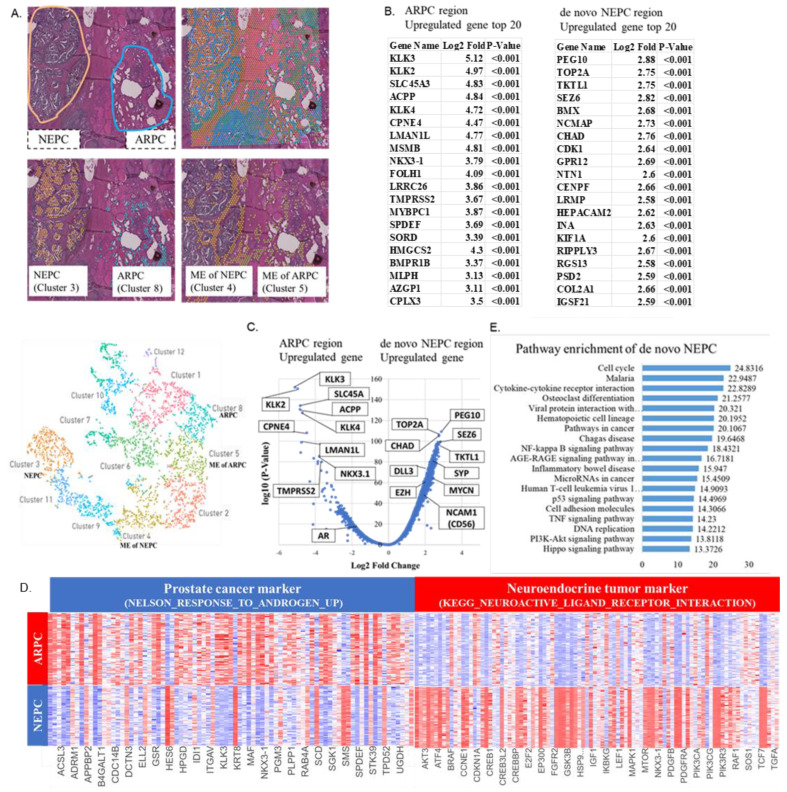
(**A**) Spatial gene expression analysis (CytAssist Visium) classifies the cells in the prostate tissue into 12 clusters. Of the 12 clusters, cluster 8 matches the androgen receptor pathway-positive adenocarcinoma of the prostate (ARPC) region, and cluster 3 matches the neuroendocrine prostate carcinoma (NEPC) region. (**B**) The top 20 most highly expressed genes in the ARPC and de novo NEPC regions. (**C**) The volcano plot demonstrating highly expressed genes in the ARPC and de novo NEPC region. (**D**) Heat map analysis of high-expression genes of the ARPC region and the de novo NEPC region. As markers defining ARPC and NEPC, gene sets accessible via GSEA (Gene Set Enrichment Analysis) are used. “NELSON_RESPONSE_TO_ANDROGEN_UP” is used as a gene set of the ARPC marker, and “KEGG_NEUROACTIVE_LIGAND_ RECEPTOR_INTERACTION” is used as a gene set of the NEPC marker. (**E**) Pathway enrichment analysis showing cell cycle activation, TNF signaling, and PI3K-Akt signaling in the de novo NEPC region.

The HE specimen showed that the NEPC region is separated from the ARPC region with intercalated noncancerous tissue between them, suggesting that ARPC and de novo NEPC occurred simultaneously and ectopically in the same prostate. Spatial gene expression analysis (CytAssist Visium) classified the cells in the prostate tissue into 12 clusters. Of the 12 clusters, cluster 8 matched the ARPC region, and cluster 3 matched the NEPC region (Figure 1A).

The 20 most highly expressed genes in the ARPC and de novo NEPC regions are shown in Figure 1B. The expression of AR signature genes such as KLK2, KLK3, and ACPP was higher in the ARPC region than in the de novo NEPC region. Meanwhile, the expression of NE signature genes such as PEG10 and TKTL1 was higher in the de novo NEPC region than in the ARPC region (Figure 1B). The volcano plot in Figure 1C shows the highly expressed genes in the ARPC and de novo NEPC regions. Similarly, heat map analysis showed predominantly high expression of AR signature genes in the ARPC region and NEPC signature genes in the de novo NEPC region (Figure 1D). Pathway enrichment analysis additionally revealed cell cycle activation, TNF signaling, and PI3K-Akt signaling in the de novo NEPC region (Figure 1E).

### 2.2. Visualization of the Expression of AR Signature Genes, NE Signature Genes, and Other Genes in the Tissue

**Figure 2:**
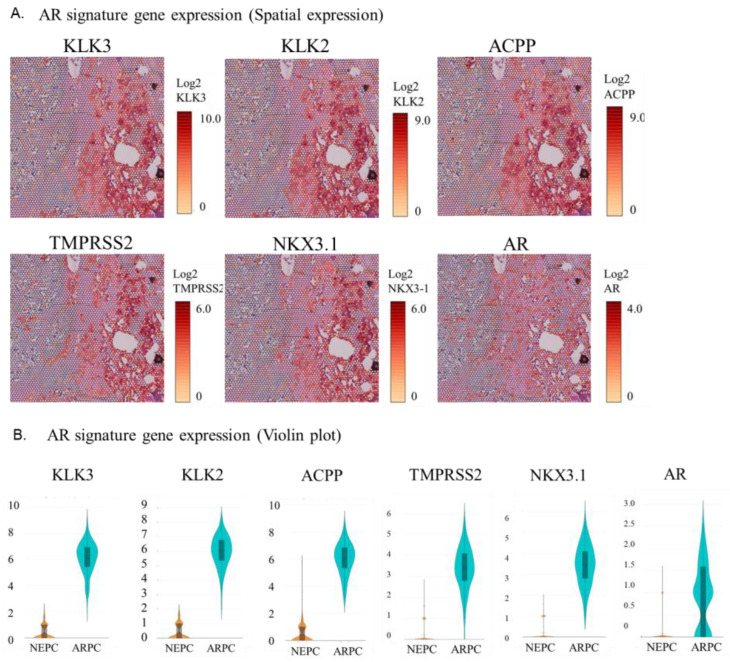

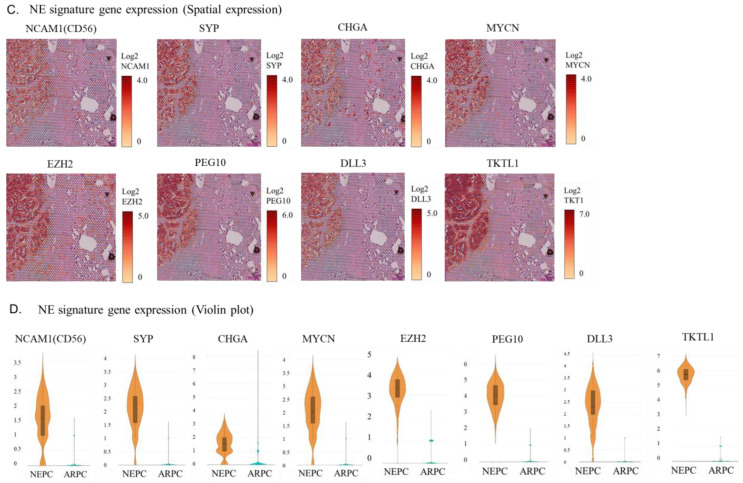
(**A**) Spatial analysis of gene expression of a group of AR signature genes. (**B**) Violin plot of gene expression of a group of AR signature genes. (**C**) Spatial analysis of gene expression of a group of NE signature genes. (**D**) Violin plot of gene expression of a group of NE signature genes.

The spatial analysis of gene expression and the violin plot showed that a group of AR signature genes including KLK2, KLK3, ACPP, TMPRSS2, NKX3.1, and AR showed high expression in the ARPC region and very low expression in the de novo NEPC region (Figure 2A,B). Similarly, the spatial analysis of gene expression and the violin plot showed that NE signature genes such as NCAM1 (CD56), SYP, CHGA, MYCN, EZH2, PEG10, DLL3, and TKTL1 were highly expressed in the de novo NEPC region but showed very low expression in the ARPC region (Figure 2C,D).

**Figure 3:**
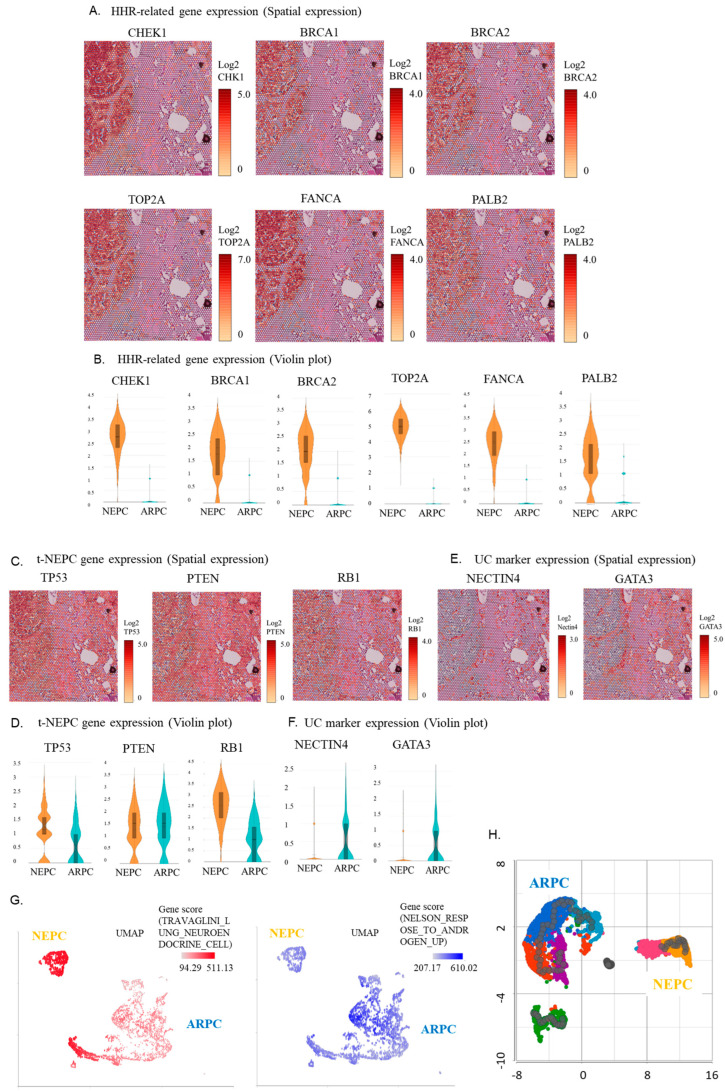
(**A**) Spatial analysis of gene expression of a group of HRR-related genes. (**B**) Violin plot of gene expression of a group of HRR-related genes. (**C**) Spatial analysis of gene expression of a group of treatment-induced neuroendocrine prostate carcinoma (t-NEPC)-related genes. (**D**) Violin plot of gene expression of a group of t-NEPC-related genes. (**E**) Spatial analysis of gene expression of a group of urothelial carcinoma marker genes. (**F**) Violin plot of gene expression of a group of urothelial carcinoma marker genes. (**G**) UMAP analysis indicating that the de novo NEPC region predominantly expresses the neuroendocrine tumor signature gene, while the ARPC region predominantly expresses the androgen response gene, clearly distinguishing the two. (**H**) Trajectory analysis showing no continuity in the genetic lineage of the ARPC and the de novo NEPC region, suggesting that they arise separately rather than via transdifferentiation.

Additionally, spatial analysis and the violin plot of gene expression showed that homologous recombination repair (HRR)-related genes such as CHEK1, BRCA1, BRCA2, TOP2A, FANCA, and PALB2 were highly expressed in the de novo NEPC region but very lowly expressed in the ARPC region (Figure 3A,B).

Meanwhile, spatial analysis of gene expression and the violin plot showed that the expressions of TP53, PTEN, and RB1, as markers of treatment-induced NEPC (t-NEPC), were similar between the de novo NEPC and ARPC regions (Figure 3C,D). This contrasted with the expectation that their expression would be lower in the de novo NEPC region than in the ARPC region. High expression of NECTIN4 or GATA3 in de novo NEPC regions is characteristic of urothelial carcinoma. However, spatial gene expression analysis and the violin plot showed no elevated expression of NECTIN4 or GATA3 in de novo NEPC regions (Figure 3E,F). Uniform Manifold Approximation and Projection (UMAP) analysis indicated that the de novo NEPC region expressed the neuroendocrine tumor signature gene, while the ARPC region predominantly expressed the androgen response gene, clearly distinguishing the two (Figure 3G). Trajectory analysis showed no continuity in the genetic lineage of ARPC and de novo NEPC, suggesting that they arose separately rather than via transdifferentiation (Figure 3H).

### 2.3. Spatial Gene Expression Analysis within the Tumor Microenvironment around ARPC and De Novo NEPC Cells

**Figure 4:**
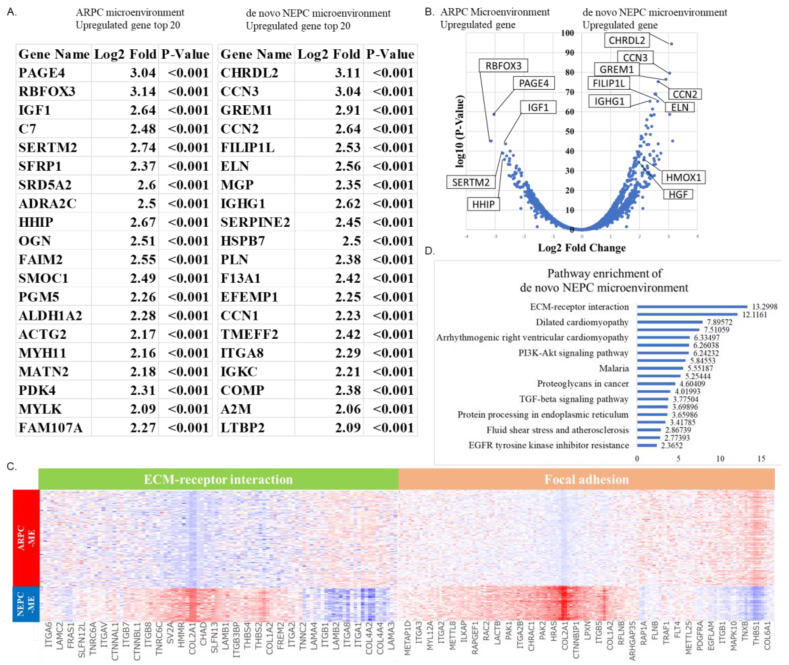
(**A**) The top 20 most highly expressed genes in the TME-ARPC and TME-de novo NEPC. (**B**) The volcano plot demonstrating the distribution of highly expressed genes in the TME-ARPC and the TME-de novo NEPC regions. (**C**) Heat map analysis of highly expressed genes in the TME-ARPC and TME-de novo NEPC regions. (**D**) Pathway enrichment analysis showing activation of ECM-receptor interaction signaling and focal adhesion signaling in the de novo NEPC region.

Of the 12 clusters, cluster 5 coincided with the TME region of the ARPC region and cluster 4, with the TME region of the de novo NEPC region (Figure 1A). The top 20 genes highly expressed in the tumor microenvironment region of ARPC (TME-ARPC) and de novo NEPC (TME-de novo NEPC) are shown in Figure 4A. The expression of RBFOX3, PAGE4, and SERTM2 were higher in the TME-ARPC region than in the TME-de novo NEPC region. Meanwhile, the expression of CHRDL2, CCM3, GREM1, and ELN were higher in the TME-de novo NEPC region than in the TME-ARPC region (Figure 4A). The volcano plot demonstrating the distribution of highly expressed genes in the TME-ARPC and the TME-de novo NEPC regions is shown in Figure 4B. Heat map analysis also showed differences in gene expression between the TME-ARPC and TME-NEPC (Figure 4C). Pathway enrichment analysis showed activation of ECM-receptor interaction signaling and focal adhesion signaling in the de novo NEPC region (Figure 4D).

**Figure 5:**
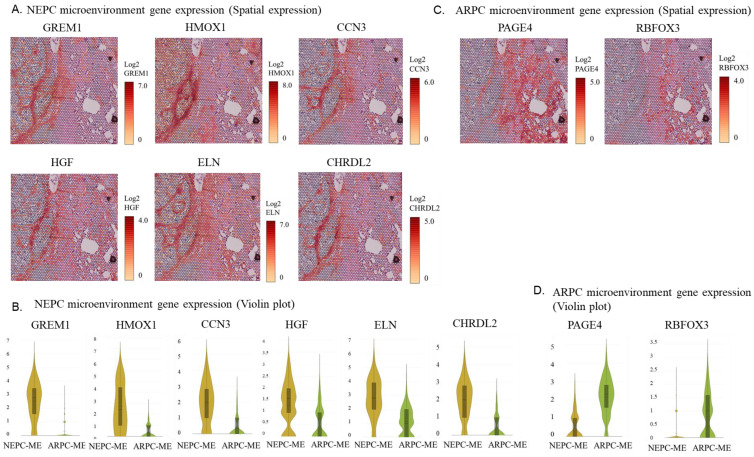
(**A**) Spatial analysis of gene expression of highly expressed genes in the tumor microenvironment around ARPC (TME-ARPC). (**B**) Violin plot of highly expressed genes in TME-ARPC. (**C**) Spatial analysis of highly expressed genes in the tumor microenvironment around NEPC (TME-de novo NEPC). (**D**) Violin plot of highly expressed genes in TME-de novo NEPC.

Spatial analysis of gene expression and violin plots revealed that fibrosis markers such as GREM1, HMOX1, CCN3, HGF, ELN, and CHRD2 were highly expressed in the TME-de novo NEPC region and lowly expressed in the de novo NEPC region (Figure 5A,B). Spatial analysis and violin plots of gene expression showed that genes associated with neuronal differentiation, such as PAGE4 and RBFOX3, were highly expressed in the TME-ARPC region and lowly expressed in the TME-NEPC region (Figure 5C,D).

## 3. Discussion

**Figure 6:**
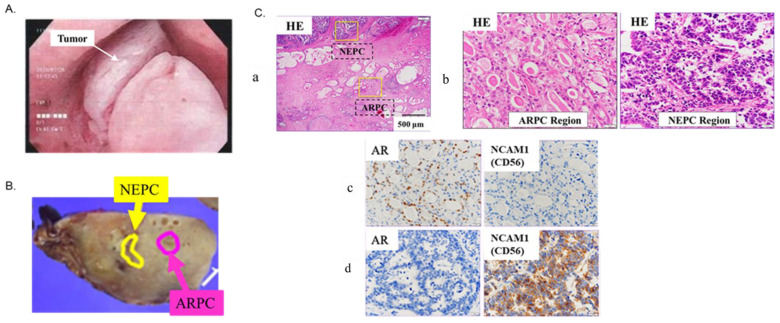
Case Presentation. (**A**) Preoperative bladder scope showing the non-papillary tumor. (**B**) The resected specimen. Pathological findings show NEPC pT4N1M0 and ARPC pT2aN0M0. The neuroendocrine prostate carcinoma (NEPC) area is separated from the androgen receptor pathway-positive adenocarcinoma of the prostate (ARPC) area. (**C**) Immunohistochemistry reveals the presence of ARPC and NEPC regions within the same section. (**a**) Hematoxylin-eosin (HE) staining (containing both the ARPC and NEPC regions) in the low-power field. (**b**) HE staining of ARPC and NEPC regions in the high-power field. (**c**) ARPC region; AR (+)/NCAM1 (CD56) (−). (**d**) NEPC region; AR (−)/NCAM1 (CD56) (+).

The present study reports an extremely rare case of concurrent ARPC and de novo NEPC diagnosed after cystoprostatectomy. The neuroendocrine carcinoma of the prostate had invaded the prostatic urethra and was not an intra-urethral recurrence of bladder cancer. ARPC and NEPC occurring within the same prostate were considered to have different origins as there was no continuity between them. Given that the NEPC region is differentiated from the ARPC area with intercalated noncancerous tissue between them and hence has no mutual migration, it is thought that ARPC and de novo NEPC has occurred ectopically and simultaneously in the same prostate (Figure 6B,C), representing intra-nodular heterogeneity [5].

Numerous single-cell analysis studies have been conducted to elucidate the mechanism of adeno-to-neuroendocrine transdifferentiation. Single cell RNA-seq by Wang et al. showed that a hierarchical transcription factor network regulated by ASCL1 and FOXA2 and selective regulation by NKX2-2, POU3F2, and SOX2 could induce transdifferentiation [6]. In addition, Han et al. analyzed mouse prostate cancer samples using single-cell multiomics analyses and reported that FOXA2 coordinated prostate cancer adeno-to-neuroendocrine lineage transition [7]. However, the current research is unique in that it is not a study on t-NEPC, but a very rare case in which the spatial gene expression of de novo NEPCs adjacent to ARPCs is the subject of analysis. The visual analysis of the differences in gene expression patterns of the microenvironments by spatial transcriptomics is a novel approach.

As shown in Figure 1 and Figure 2, AR signature genes were highly expressed in the ARPC region, while NE signature genes were highly expression in the de novo NEPC region, as initially expected. KLK genes are known to be highly expressed in ARPC, and KLK2, KLK3, and KLK11 were also highly expressed in ARPC in the current case. However, KLK6 and KLK12 were highly expressed in the de novo NEPC region, and their expression was decreased in ARPC. KLK6 and KLK12 are reported to be markers associated with highly aggressive prostate cancer [8,9], but future studies are still needed to validate these findings. The high expression of ERG, which is derived from the TMPRSS2-ERG fusion gene and is upregulated in response to androgen, is reported to be associated with malignancy [10], but the high ERG expression was found in neither the ARPC region nor NEPC region in the present case. 

In the present case, ARPC did not have very strong gene expression of AR, and de novo NEPC did not have very strong gene expression of CHGA. The expressions of SYP, CHGA, and NCAM1 are commonly used in the pathological diagnosis of NEPC, but it is rare for all three to be positive. Most cases are diagnosed by elevation of at least one NE marker. In the present case, only NCAM1 (CD56) was positive on immunohistochemistry. Among the top upregulated genes in NEPC, SYP ranks 57th; CHGA, 312nd; and NCAM1, 482nd. These genes are not upregulated gene in ARPC. Similarly, AR alone is insufficient for pathological diagnosis because its expression level varies. In the current case, it is the 157th most upregulated gene in the ARPC region but is not upregulated in the NEPC region.

The present case shows an elevated expression of PEG10, which is reported to be highly expressed in NEPC. Akamatsu et al. reported that PEG10 is suppressed in the adaptive response to AR interference and is very highly expressed in NEPC [11]. Furthermore, TKTL1 which was upregulated in the de novo NEPC region in the present case, is an important protein that promotes invasion and metastasis and is highly expressed in neuroendocrine tumors (NETs) [12]. Thus, it is not surprising that it is upregulated in the de novo NEPC in the current case. DLL3, a member of the NOTCH signaling pathway, is expressed in 76.7% of NEPCs and is also highly expressed in the current case. In an in vivo model, treatment with SC16LD6.5 elicited a complete response in DLL3-positive tumors but not in DLL3-negative tumors, making it an attractive modality for NEPC treatment [13]. Meanwhile, the expressions of TP53, RB1, and PTEN, which are known to be significantly downregulated in t-NEPC, showed similar expression patterns in the ARPC and de novo NEPC regions. The mechanism of transdifferentiation into t-NEPCs is multifactorial and requires alterations in tumor suppressor genes, such as TP53, RB1, and PTEN [14,15,16,17,18,19]. Considering that the genomic information of NEPC is similar to that of castration-resistant prostate cancer (CRPC) [10], most t-NEPCs are thought to arise from CRPC [20]. This supports our results that the de novo NEPC region in the present case is not differentiated from the ARPC region, but could be developed by other mechanism. However, further investigation requires detailed analysis by RNA-seq because the CytAssist Visium cannot detect transdifferentiation. In addition, the absence of elevated markers of urothelial carcinoma such as NECTIN4 and GATA3 (shown in Figure 3E,F) in the present case suggests that the malignancy is unlikely to be derived from bladder cancer.

In the present case, the HRR-related genes showed increased expression in the de novo NEPC region. HRR genes have been recently suggested to be involved in the pathogenesis of NETs such as small cell lung cancer and pancreatic NETs [21]. A possible mechanism of NET development by HRR genes is the acquisition of the ability to grow unlimitedly due to failure of efficient and accurate DNA repair [22]. However, to our best knowledge, the involvement of HRR genes in the pathogenesis of de novo NEPC has not been reported. Meanwhile, a high rate of HRR gene mutations has been reported in t-NEPC, which is similar to the mutation profile in advanced prostate cancer [23]. This finding is novel because it suggests that the HRR gene may be involved in the pathogenesis of de novo NEPC. It also suggests that PARP inhibitors are likely to be effective for de novo NEPC. It has been previously reported that TOP2A accumulation is induced by SPOP mutation and contributes to prostate cancer progression from the accumulation of DNA damage, and etoposide could be effective for SPOP mutation prostate cancer [24]. In the present case, although TOP2A was upregulated, the SPOP gene was not mutated.

The role of the TME in tumor progression cannot be ignored [25]. In the present case, the ECM-receptor interaction and focal adhesion pathways were the most enriched functional annotations in genes upregulated in the TME-de novo NEPC region. These pathways are involved in the interaction between tumor cells and the extracellular matrix, cell adhesion, cell motility, morphological changes, cell growth, proliferation, invasion, and metastasis, suggesting its high potential for promoting tumor invasiveness and metastasis. Rbfox3 plays an important role in the regulation of TGF-β-induced epithelial-mesenchymal transition (EMT), and depletion of RBFOX3 increases the expression of a group of proteins associated with EMT such as E-cadherin and Claudin-1, which enhance mesenchymal morphology. In the present case, the elevated expression of RBFOX3 in TME-ARPCs and the depletion of RBFOX3 in TME-de novo NEPCs may reflect the tumor progression potential of de novo NEPCs [26]. The SFRTM2 gene plays a role in regulating the abnormal activation of Wnt signaling in gastric and colorectal cancer [27], and the differentiation of ARPCs into t-NEPCs is associated with the activation of Wnt signaling by therapeutic stimuli [28]. Hence, the upregulation of the SFRP gene in TME-ARPCs and its downregulation in TME-de novo NEPCs in the present case is reasonable.

HGF, HMOX1, ELN, and GREM1 genes associate with stromal fibrosis and contribute to malignancy through the proliferation of cancer-associated fibroblasts (CAFs) in various kinds of cancers [29,30,31,32]. De Hosson et al. reported that neuroendocrine tumors originating from the lung, pancreas, and stomach have very low levels of T-cells and high levels of CAFs, which are involved in maintaining an “immune cold” environment and reduce the efficacy of immune checkpoint inhibitors [33]. As immune cell access is impaired, therapeutic approaches that improve the TME and enhance the efficacy of immune checkpoint inhibitors are important. Although the analysis of only one case did not allow us to determine the detailed mechanism of development, it is worth mentioning that the downregulation of Rbfox3 and SFRTM2 and upregulation of CAF markers (i.e., HGF, HMOX1, ELN, and GREM1) suggested the influence of the TME on de novo NEPC development. 

Spatial transcriptomics, which enabled us to maintain the pathological location information, was particularly useful in the current case, and more information was obtained. The novelty of this study is that to our best knowledge, it is the first report to visually compare gene expression of de novo NEPCs with that of adjacent ARPCs using spatial transcriptomics analysis. Furthermore, spatial transcriptomics for the visual analysis of the differences in gene expression patterns of the microenvironments is another novelty. However, the limitation of this study is that only one case was analyzed, although the data collected will be useful for a better understanding of the genetic mechanism of de novo NEPC. There still might be many factors associated with the occurrence of NEPC, and therefore, it is essential to accumulate more evidence from more cases and data. Elucidation of the pathogenesis of NEPC will lead to the development of novel treatments and may also contribute to improving the prognosis of patients with CRPC. 

## 4. Patient and Methods

The study was approved by the Institutional Review Board of Ehime University (No. 2108006, 2109014, 2205001) and was conducted according to the principles of the Declaration of Helsinki. Written informed consent was obtained from the patient for the publication of the case report and accompanying images.

### 4.1. Case Presentation

A 78-year-old man who had no family history of hereditary disease or cancer presented to our hospital with gross hematuria and underwent transurethral resection of bladder tumor (TUR-BT) for bladder tumor. A second TUR-BT was also performed after the first TUR-BT. The pathological findings revealed invasive urothelial carcinoma, G3, and pT1 with CIS. Based on the diagnosis, Bacille Calmette-Guerin (BCG) therapy was administered for non-muscle invasive urothelial carcinoma of the bladder. At 5 months after the BCG therapy, a follow-up cystoscopy revealed a neoplastic lesion in the prostatic urethra (Figure 6A), and hence, TUR was performed. Pathological findings revealed a neuroendocrine carcinoma. Positron emission tomography-computed tomography (PET-CT) demonstrated right external iliac lymph node metastasis and left obturator lymph node metastasis. Tumor marker investigation revealed neuron-specific enolase (NSE) at 15.5 ng/mL (normal value: < 16.3 ng/mL), pro-gastrin-releasing peptide (proGRP) at 39.8 pg/mL (normal value: <81 pg/mL), and prostate-specific antigen (PSA) at 2.57 ng/mL (normal value: <4 ng/mL). Based on the diagnosis of neuroendocrine carcinoma of the prostatic urethra with lymph node metastasis, robot-assisted laparoscopic cystectomy/prostatectomy with pelvic lymphadenectomy was performed. Pathological findings of the bladder/prostate samples showed a mixed carcinoma of NEPC pT4N1M0 and ARPC pT2aN0M0, Gleason score of 4 + 4, and without urothelial carcinoma in the bladder (Figure 6B,C). Lymph node dissection revealed metastases of neuroendocrine tumor origin in the left internal and right external iliac lymph nodes.

The lymph node specimens showed metastases of pure neuroendocrine carcinoma in the left internal and right external iliac lymph nodes. Four courses of etoposide + cis-platinum (EP) were administered as adjuvant chemotherapy. The patient then had superior mediastinal lymph node metastasis and hence was started on pembrolizumab. The patient maintained stable disease for 9 months. 

### 4.2. Spatial Transcriptomics (CytAssist Visium)

Formalin-fixed, paraffin-embedded (FFPE) samples passing the RNA quality control (DV200 > 50%) were used for spatial transcriptomic construction and sequencing. The tissue was prepared according to the Visium CytAssist Spatial Gene Expression for FFPE-Tissue Preparation Guide (CG000518, 10× Genomics, Pleasanton, CA, USA). The sequencing was performed by the Research Institute for Microbial Diseases at Osaka University. Libraries were sequenced on an MGI DNBSEQ-G400RS (MGI Tech Co., Shenzhen, China). The Space Ranger pipeline v2022.0705.1 (10× Genomics, Pleasanton, CA, USA) and the GRCh38-2020-A reference were used to process FASTQ files. The sequence results were guaranteed to be accurate as follows:

1. Number of spots under tissue: 4397; mean reads per spot: 74,832; median genes per spot: 7195; number of reads: 329,038,054; valid barcodes: 94.0%; valid UMIs: 99.9%; sequencing saturation: 48.0%
2. UMAP and violin plots were run and plotted using Loupe Browser (10× genomics, Pleasanton, CA, USA). Trajectory analysis and pathway enrichment analysis were performed and plotted using Partek flow software (Partek Incorporated, Chesterfield, MO, USA).

## 5. Conclusions

The findings of spatial gene expression analysis in a patient with coexisting ARPC and de novo NEPC is reported. The causative factors for the incidence of NEPC remain unknown. The accumulation of cases and basic data will enable research and lead to the development of novel treatments for NEPC and improve the prognosis of patients with CRPC. The open data analyzed in this study can further our understanding of the genetics of NEPC.

## Data Availability

The raw and processed data of spatial transcriptomics generated in this study are openly available in GEO at https://www.ncbi.nlm.nih.gov/geo/, (accessed on 13 May 2023) GEO number {GSE230282}.

## References

[1] Chen R., Dong X., Gleave M. (2018). Molecular Model for Neuroendocrine Prostate Cancer Progression. BJU Int..

[2] Sargos P., Ferretti L., Gross-Goupil M., Orre M., Cornelis F., Henriques de Figueiredo B., Houédé N., Merino C., Roubaud G., Dallaudiére B. (2014). Characterization of Prostate Neuroendocrine Cancers and Therapeutic Management: A Literature Review. Prostate Cancer Prostatic Dis..

[3] Patel G.K., Chugh N., Tripathi M. (2019). Neuroendocrine Differentiation of Prostate Cancer-An Intriguing Example of Tumor Evolution at Play. Cancers.

[4] Chen J., Shi M., Chuen Choi S.Y., Wang Y., Lin D., Zeng H., Wang Y. (2023). Genomic alterations in neuroendocrine prostate cancer: A systematic review and meta-analysis. BJUI Compass.

[5] Miyagi Y., Sasaki T., Fujinami K., Sano J., Senga Y., Miura T., Kameda Y., Sakuma Y., Nakamura Y., Harada M. (2010). ETS Family-associated Gene Fusions in Japanese Prostate Cancer: Analysis of 194 Radical Prostatectomy Samples. Mod. Pathol..

[6] Hong D., Dong B., Wang Y., Huang H., Zhang W., Lian B., Ji B., Shi H., Qu M., Gao X. (2022). Single-Cell Transcriptional Regulation and Genetic Evolution of Neuroendocrine Prostate Cancer. iScience.

[7] Han M., Li F., Zhang Y., Dai P., He J., Li Y., Zhu Y., Zheng J., Huang H., Bai F. (2022). FOXA2 Drives Lineage Plasticity and KIT Pathway Activation in Neuroendocrine Prostate Cancer. Cancer Cell.

[8] Lose F., Batra J., O’Mara T., Fahey P., Marquart L., Eeles R.A., Easton D.F., Al Olama A.A., Kote-Jarai Z., Guy M. (2013). Common Variation in Kallikrein Genes KLK5, KLK6, KLK12, and KLK13 and Risk of Prostate Cancer and Tumor Aggressiveness. Urol. Oncol..

[9] Korbakis D., Soosaipillai A., Diamandis E.P. (2017). Study of Kallikrein-related Peptidase 6 (KLK6) and Its Complex with α1-Antitrypsin in Biological Fluids. Clin. Chem. Lab. Med..

[10] Lotan T.L., Gupta N.S., Wang W., Toubaji A., Haffner M.C., Chaux A., Hicks J.L., Meeker A.K., Bieberich C.J., De Marzo A.M. (2011). ERG Gene Rearrangements Are Common in Prostatic Small Cell Carcinomas. Mod. Pathol..

[11] Akamatsu S., Wyatt A.W., Lin D., Lysakowski S., Zhang F., Kim S., Tse C., Wang K., Mo F., Haegert A. (2015). The Placental Gene PEG10 Promotes Progression of Neuroendocrine Prostate Cancer. Cell Rep..

[12] Modlin I.M., Drozdov I., Alaimo D., Callahan S., Teixiera N., Bodei L., Kidd M. (2014). A Multianalyte PCR Blood Test Outperforms Single Analyte ELISAs (Chromogranin A, Pancreastatin, Neurokinin A) for Neuroendocrine Tumor Detection. Endocr. Relat. Cancer.

[13] Puca L., Gavyert K., Sailer V., Conteduca V., Dardenne E., Sigouros M., Isse K., Kearney M., Vosoughi A., Fernandez L. (2019). Delta-like Protein 3 Expression and Therapeutic Targeting in Neuroendocrine Prostate Cancer. Sci. Transl. Med..

[14] Yamada Y., Beltran H. (2021). Clinical and Biological Features of Neuroendocrine Prostate Cancer. Curr. Oncol. Rep..

[15] Merkens L., Sailer V., Lessel D., Janzen E., Greimeier S., Kirfel J., Perner S., Pantel K., Werner S., von Amsberg G. (2022). Aggressive Variants of Prostate Cancer: Underlying Mechanisms of Neuroendocrine Transdifferentiation. J. Exp. Clin. Cancer Res..

[16] Brennen W.N., Zhu Y., Coleman I.M., Dalrymple S.L., Antony L., Patel R.A., Hanratty B., Chikarmane R., Meeker A.K., Zheng S.L. (2021). Resistance to Androgen Receptor Signaling Inhibition Does Not Necessitate Development of Neuroendocrine Prostate Cancer. JCI Insight.

[17] Akamatsu S., Inoue T., Ogawa O., Gleave M.E. (2018). Clinical and Molecular Features of Treatment-related Neuroendocrine Prostate Cancer. Int. J. Urol..

[18] Aggarwal R., Huang J., Alumkal J.J., Zhang L., Feng F.Y., Thomas G.V., Weinstein A.S., Friedl V., Zhang C., Witte O.N. (2018). Clinical and Genomic Characterization of Treatment-emergent Small-cell Neuroendocrine Prostate Cancer: A Multi-institutional Prospective Study. J. Clin. Oncol..

[19] Beltran H., Rickman D.S., Park K., Chae S.S., Sboner A., MacDonald T.Y., Wang Y., Sheikh K.L., Terry S., Tagawa S.T. (2011). Molecular Characterization of Neuroendocrine Prostate Cancer and Identification of New Drug Targets. Cancer Discov..

[20] Beltran H., Prandi D., Mosquera J.M., Benelli M., Puca L., Cyrta J., Marotz C., Giannopoulou E., Chakravarthi B.V.S.K., Varambally S. (2016). Divergent Clonal Evolution of Castration-resistant Neuroendocrine Prostate Cancer. Nat. Med..

[21] Sen T., Gay C.M., Byers L.A. (2018). Targeting DNA Damage Repair in Small Cell Lung Cancer and the Biomarker Landscape. Transl. Lung Cancer Res..

[22] Hanahan D., Weinberg R.A. (2011). Hallmarks of Cancer: The Next Generation. Cell.

[23] Robinson D., Van Allen E.M., Wu Y.M., Schultz N., Lonigro R.J., Mosquera J.M., Montgomery B., Taplin M.-E., Pritchard C.C., Attard G. (2015). Integrative Clinical Genomics of Advanced Prostate Cancer. Cell.

[24] Watanabe R., Maekawa M., Hieda M., Taguchi T., Miura N., Kikugawa T., Saika T., Higashiyama S. (2020). SPOP is Essential for DNA-Protein Cross-link Repair in Prostate Cancer Cells: SPOP-dependent Removal of Topoisomerase 2A from the Topoisomerase 2A-DNA Cleavage Complex. Mol. Biol. Cell.

[25] Brady L., Nelson P.S. (2022). RISING STARS: Heterogeneity and the Tumor Microenvironment in Neuroendocrine Prostate Cancer. J. Endocrinol..

[26] Kim Y.E., Kim J.O., Park K.S., Won M., Kim K.E., Kim K.K. (2016). Transforming Growth Factor-β-induced RBFOX3 Inhibition Promotes Epithelial-mesenchymal Transition of Lung Cancer Cells. Mol. Cells.

[27] Zhao H., Ming T., Tang S., Ren S., Yang H., Liu M., Tao Q., Xu H. (2022). Wnt Signaling in Colorectal Cancer: Pathogenic Role and Therapeutic Target. Mol. Cancer.

[28] Sun Y., Campisi J., Higano C., Beer T.M., Porter P., Coleman I., True L., Nelson P.S. (2012). Treatment-induced Damage to the Tumor Microenvironment Promotes Prostate Cancer Therapy Resistance through WNT16B. Nat. Med..

[29] Ding X., Xi W., Ji J., Cai Q., Jiang J., Shi M., Yu Y., Zhu Z., Zhang J. (2018). HGF Derived from Cancer-associated Fibroblasts Promotes Vascularization in Gastric Cancer via PI3K/AKT and ERK1/2 Signaling. Oncol. Rep..

[30] Luu Hoang K.N., Anstee J.E., Arnold J.N. (2021). The Diverse Roles of Heme Oxygenase-1 in Tumor Progression. Front. Immunol..

[31] Liu T., Zhou L., Li D., Andl T., Zhang Y. (2019). Cancer-associated Fibroblasts Build and Secure the Tumor Microenvironment. Front. Cell Dev. Biol..

[32] Ren J., Smid M., Iaria J., Salvatori D.C.F., van Dam H., Zhu H.J., Martens J.W.M., Dijke P.T. (2019). Cancer-associated Fibroblast-derived Gremlin 1 Promotes Breast Cancer Progression. Breast Cancer Res..

[33] De Hosson L.D., Takkenkamp T.J., Kats-Ugurlu G., Bouma G., Bulthuis M., de Vries E.G.E., Van Faassen M., Kema I.P., Walenkamp A.M.E. (2020). Neuroendocrine Tumours and Their Microenvironment. Cancer Immunol. Immunother..

